# The 164 K, 165 K, and 167 K residues of VP1 are vital for goose parvovirus proliferation in GEFs based on PCR-based reverse genetics system

**DOI:** 10.1186/s12985-019-1237-2

**Published:** 2019-11-14

**Authors:** Peng Liu, Liqin Yang, Jingyue Zhang, Tao Wang, Yuanyuan Wu, Mingshu Wang, Renyong Jia, Dekang Zhu, Mafeng Liu, Xinxin Zhao, Qiao Yang, Ying Wu, Shaqiu Zhang, Yunya Liu, Yanling Yu, Ling Zhang, Leichang Pan, Shun Chen, Anchun Cheng

**Affiliations:** 1 Research Center of Avian Disease, College of Veterinary Medicine, Sichuan Agricultural University, Wenjiang District, Chengdu City, 611130 Sichuan Province China; 2 Institute of Preventive Veterinary Medicine, College of Veterinary Medicine, Sichuan Agricultural University, No. 211 Huimin Road, Wenjiang District, Chengdu City, 611130 Sichuan Province China; 3 Key Laboratory of Animal Disease and Human Health of Sichuan Province, Wenjiang District, Chengdu City, 611130 Sichuan Province China

**Keywords:** GPV, Infectious clone, NLS, Proliferation, GEF

## Abstract

**Background:**

Goose parvovirus (GPV) is the etiological agent of Derzsy’s disease and is fatal for gosling. Research on the molecular basis of GPV pathogenicity has been hampered by the lack of a reliable reverse genetics system. At present, the GPV infectious clone has been rescued by transfection in the goose embryo, but the growth character of it is unclear in vitro.

**Methods:**

In this study, we identified the full-length genome of GPV RC16 from the clinical sample, which was cloned into the pACYC177, generating the pIRC16. The recombinant virus (rGPV RC16) was rescued by the transfection of pIRC16 into goose embryo fibroblasts (GEFs). The rescued virus was characterized by whole genome sequencing, indirect immunofluorescence assays (IFA) and western blot (WB) using rabbit anti-GPV Rep polyclonal antibody as the primary antibody. Previously, we found the 164 K, 165 K, and 167 K residues in the 160YPVVKKPKLTEE171 are required for the nuclear import of VP1 (Chen S, Liu P, He Y, et al. Virology 519:17–22). According to that, the GPV infectious clones with mutated K164A, K165A, or K167A in VP1 were constructed, rescued and passaged.

**Results:**

The rGPV RC16 has been successfully rescued by transfection of pIRC16 into the GEFs and can proliferate in vitro. Furthermore, the progeny virus produced by pIRC16 transfected cells was infectious in GEFs. Moreover, mutagenesis experiments showed that the rGPV RC16 with mutated 164 K, 165 K and 167 K in VP1 could not proliferate in GEFs based on the data of IFA and WB in parental virus and progeny virus.

**Conclusions:**

The rGPV RC16 containing genetic maker and the progeny virus are infectious in GEFs. The 164 K, 165 K, and 167 K of VP1 are vital for the proliferation of rGPV RC16 in vitro.

## Background

Goose parvovirus (GPV) is known as Derzsy’s disease [1]. It can infect the goslings and muscovy duck, showing lethargy, ataxia, weight loss, palpebral swellings, dysphagia, and anorexia, and can cause high mortality, especially in goslings [2]. However, muscovy duck parvovirus (MDPV) only can infect muscovy duck and show clinical signs [3]. Recently, a novel GPV (NGPV) has been identified and only can infect duck, showing low morality, beak atrophy and dwarfism syndrome [4, 5]. GPV, MDPV and NGPV are the *Dependoparvovirus* genus of the *Parvovirinae* subfamily within the *Parvoviridae* family.

GPV is a single-stranded DNA virus without envelop protein, and the entire genome is about 5.1 kb in length, which contains the inverted terminal repeats (ITR) at both genomic terminus and two major open reading frames (ORF) [6]. The ITR contains the signal of replication and encapsidation [7]. And a GTTC element within the GPV ITR was found that it can be strongly bound by GPV replication protein 1 (Rep1) and identified as the GPV replication origin [8]. The left ORF encodes the non-structural protein required for both replication of viral genome and regulation of capsid gene expression [9, 10], and the right ORF encodes three capsid proteins (VP1/2/3) which share common region of C-terminus [11]. The capsid is composed of VP1, 2, 3 and the VP3 is the major portion of the entire capsid [12]. Our previously data indicated that the basic region (BR, 160YPVVKKPKLTEE171) was identified as a classical nuclear localization signal (NLS) in the VP1 N-terminus and the 164 K, 165 K and 167 K played a key role [13]. This NLS is important for the translocation of GPV VP1 into the nucleus, however, its role in GPV life-cycle hasn’t been studied yet.

In this study, we have successfully cloned and sequenced the full-length genome of a virulent GPV RC16 strain. Theentire genome of GPV RC16 has been cloned into the pACYC177 referred as pIRC16. Then the infectious virions were successfully rescued by transfecting goose embryo fibroblasts (GEFs) with pIRC16. Finally, the virus from transfection of infectious clone with mutated 164 K, 165 K, and 167 K can’t proliferate in GEFs, indicating the NLS key amino acid of VP1 is vital for rGPV RC16 proliferation. This work will provide a foundation for future studies of the infection and pathogenic mechanism of GPV.

## Materials and methods

### Cells and virus

GEFs were separated from the 9-day goose embryo and grown in Dulbecco’s modified Eagle’s medium containing 10% fetal bovine serum (Gibco Life Technologies, Shanghai, China) at 37 °C in an atmosphere with 5% CO_2_.

The GPV RC16 strain was isolated from the liver of an ill goose [14] and the viral DNA was extracted by using TIANamp Virus DNA/RNA Kit (Tiangen, Beijing) according to the protocol.

### Sequence amplification of GPV RC16 strain

Three pairs of primers (Table 1) were designed to amplify the GPV Rep1 and GPV VP1, respectively. The PCR products were analyzed by electrophoresis in a 1% agarose gel. The DNA fragments from PCR were extracted by using the TaKaRa MiniBEST DNA Fragment Purification Kit Ver.4.0 (Takara, Dalian, China). According to the sequence of GPV YZ99–6, we designed three pairs of primers (Table 1) to amplify the right and left ITR. As expected, the ITR of GPV RC16 shown highly identity with the GPV YZ99–6. The fragments were cloned into the pMD19-T (Takara, Dalian, China) by TA clone and named pMD-GPV 1–187, pMD-GPV 188–412, pMD-GPV 412–2492, pMD-GPV 2493–4015, pMD-GPV 4016–4863 and pMD-GPV 4864–5046, respectively, which were directly sequenced at TSINGKE Biological Technology (Chengdu, Sichuan, China).

**Table 1:** The oligonucleotide primer used for amplification of GPV RC16 genome in this study

Primer	The sequence (5′-3′)
A1F	TCATTGGAGGGTTCGTTCGTTC
A1R	CATGCGCGCGGTCAACCTAACAGCCG
A2F	CGCGCGGTCAGCCCAATAGTTAAGCC
A2R	CTTCCTGGCGCGCAAAATATC
A3F	GATATTTTGCGCGCCAGGAAG
A3R	GAGGGGCTCCAGCTTTCAGATTCC
A4F	CGGAATCTGAAAGCTGGAGC
A4R	CTAAAATATTTTGGGCTGGGATGC
A5F	TCAGCTACTCACACAGAAG
A5R	CATGCGCGCGGTCAGCCCAATAG
A6F	CGCGCGGTCAACCTAACAGCCGG
A6R(A1F)	TCATTGGAGGGTTCGTTCGTTC

### Construction of GPV RVC16 infectious clone

#### Construction of the pAC KS vector

To facilitate cloning, a linker of 5-KpnI-XhoI-SalI-HindIII-EcoRV-EcoRI-SmaI-3 was inserted into the MCS of pACYC177 between *Nhe*I and *Xam*I generating pAC KS vector.

#### Construction of the full-length GPV RC16 clone

The DNA fragment BstZ17I-NheI-nt 1–187-XhoI-HindIII (containing the sequence of GPV 1–187, the primers as shown in Table 2) was amplified from pMD-GPV 1–187 and cloned into the pAC KS between *Nhe*I and *Xho*I to produce pAC GPV 1–187 by using One-Step Cloning Kit (Vazyme, Nanjing, China). The second fragment nt 167–412-HindIII-EcoRV (containing the sequence of GPV 188–412) was amplified from pMD-GPV 188–412 and cloned into the pAC GPV 1–187 between *Xho*I and *Hind*III to produce pAC GPV1–412. The exogenous restriction enzyme site *Xho*I within the primer would be deleted by the One-Step Cloning Kit in the process of construction, thus the intact ITR could be obtained. The third fragment nt 392–2492-HindIII-EcoRV (containing the sequence of GPV 412–2492, a genetic maker (M: AAGCTT→GAGTTT) introduced into pMD-GPV 412–2492 before amplifying was amplified from pMD-GPV 412–2492 and cloned into pAC GPV1–412 between *Hind*III and *EcoR*V to produce pAC GPV1–2492. The fourth, fifth and sixth fragments were amplified from pMD-GPV 2493–4015, pMD-GPV 4016–4863 and pMD-GPV 4864–5046 and cloned into the plasmid sequentially. And the final construct containing the full-length GPV RC16 was designated as pIRC16. pIRC16 was amplified by PCR using the primers in Table 1, and the amplified DNA fragments were subsequently sequenced, indicating that there were no unexpected mutations other than the genetic markers.

**Table 2:** The oligonucleotide primer used for construction of GPV RC16 infectious clone in this study

Primer	The sequence (5′-3′)
pACA1F	GCCAGTATACACTCCGCTAGCTCATTGGAGGGTTCG
pACA1R	TCGATACCGTCGACCTCGAGCATGCGCGCGGTCAAC
pACA2F	GTTAGGTTGACCGCGCGCATGCGCGCGGTCAGCCC
pACA2R)	CAGGAATTCGATATCAAGCTTCTTCCTGGCGCGCAAAATATC
pACA3F	GATATTTTGCGCGCCAGGAAGTGACGTGCAATGCCAC
pACA3R	CAGGAATTCGATATCAAGCTTCTACCGGGTAGTGGTC
pACA4F	AAAGCTGGAGCCCCTCACCCAAAACCAAACCAGCAGACTCAG
pACA4R	TGGCAAAGTGGATCCCCCGGGCTGCAGGAATTCCTAAAATATTTTGGGC
pACA5F	CCAGCCCAAAATATTTTAGGTTTAGCTAAAGATC
pACA5R	TGGCAAAGTGGATCCCCCGGGCATGTGGAGCTCCAGCCCAATAGTTAAG
pACA6F	CTTAACTATTGGGCTGGACCGCGCGCATGCG
pACA6R	ATGGCAAAGTGGATCCCCCGGGTCATTGGAGGGTTCGTTCG

The region of plasmid vector was indicated by underline

### Construction of GPV RC16 defect clone with NLS site mutations

The three key amino acid sites of NLS, 164 K, 165 K and 167 K in GPV VP1 have been respectively substituted by Ala based on pIRC16. Briefly, a DNA fragment (K164A, the DNA fragments as shown in Table 3) containing GPV RC16 genome from nt 2852 to 2997 and the mutants of 2898 A to G, 2899 A to C and 2900 G to A, was synthesized and cloned into the pIRC16 between *Hind*III and *Kpn*I. The final construct, the amino acid site 164 K was changed into A within the GPV VP1, was designated pIRC16 K164A. A similar method was used for constructing pIRC16 K165A, pIRC16 K167A. Moreover, all of the key sites of NLS have been substituted by Ala generating the pIRC16 AAPA, and the 166 P has been substituted by Lys generating the pIRC16 KKRK as the experiment control.

**Table 3:** The DNA fragments used for construction of GPV RC16 infectious clone with mutated NLS site in this study

DNA fragments	The sequence (5′-3′)
K164A	GCAAAAAAAAATACAGGGAAGCTTACCGACCACTACCCGGTAGTTGCAAAGCCTAAACTTACCGAGGAAGTCAGTGCGGGAGGTGGTAGTAGTGCCGTACAAGACGGAGGAGCCACCGCGGAGGGTACCGAACCTGTGGCAGCAT
K165A	GCAAAAAAAAATACAGGGAAGCTTACCGACCACTACCCGGTAGTTAAGGCACCTAAACTTACCGAGGAAGTCAGTGCGGGAGGTGGTAGTAGTGCCGTACAAGACGGAGGAGCCACCGCGGAGGGTACCGAACCTGTGGCAGCATC
K167A	GCAAAAAAAAATACAGGGAAGCTTACCGACCACTACCCGGTAGTTAAGAAGCCTGCACTTACCGAGGAAGTCAGTGCGGGAGGTGGTAGTAGTGCCGTACAAGACGGAGGAGCCACCGCGGAGGGTACCGAACCTGTGGCAGCATC
AAPA	GCAAAAAAAAATACAGGGAAGCTTACCGACCACTACCCGGTAGTTGCAGCACCTGCACTTACCGAGGAAGTCAGTGCGGGAGGTGGTAGTAGTGCCGTACAAGACGGAGGAGCCACCGCGGAGGGTACCGAACCTGTGGCAGCATC
KKRK	GCAAAAAAAAATACAGGGAAGCTTACCGACCACTACCCGGTAGTTAAGAAGAGGAAACTTACCGAGGAAGTCAGTGCGGGAGGTGGTAGTAGTGCCGTACAAGACGGAGGAGCCACCGCGGAGGGTACCGAACCTGTGGCAGCATC

The mutated sites were indicated by underline

### Recombinant viruses rescue and passage

When GEFs monolayer cells seeded into a 6-well plate were at 70% confluence, the pIRC16 plasmid was transfected into GEFs using lipofectamine® 3000 transfection kit (Invitrogen, USA). After 84 h post-infection (PI), the cells were harvested and repeated freezing and thawing three times. The sample was blind passaged twice in GEFs. The rescued virus was named as rGPV RC16.

Similarly, when GEFs monolayer cells seeded into a 12-well plate were at 70% confluence, the pIRC16 K164A, pIRC16 K165A, pIRC16 K167A, pIRC16 AAPA and pIRC16 KKRK were respectively transfected into GEFs using lipofectamine® 3000 transfection kit, generating the recombinant virus and named rGPV RC16 K164A, rGPV RC16 K165A, rGPV RC16 K167A, rGPV RC16 AAPA and rGPV RC16 KKRK respectively. After 72 h PI, the cells were harvested and repeated freezing and thawing three times. Subsequently, the GEFs monolayer cells seeded into a 12-well plate at 70% confluence were respectively infected with all of the harvested samples. After 72 h PI, the cells were harvested to identify the expression of GPV Rep by IFA and WB.

### Immunofluorescence assay

The GEFs monolayer cells were infected with the rGPV RC16. After infection, cells were washed with 1 × PBS three times and fixed with 4% paraformaldehyde for 1 h and permeabilized with 0.2% Triton X-100 for 15 min at room temperature. The membranes were then incubated with the primary antibody (using mouse anti-GPV polyclonal antibody or rabbit anti-GPV Rep polyclonal antibody) at a dilution of 1∶200 in PBS with 1% BSA for 12 h at 4 °C. This was followed by incubation with a fluorescein isothiocyanate or tetramethylrhodamine-conjugated secondary antibody (FITC- conjugated goat anti-mouse IgG antibody or TRITC-conjugated goat anti-rabbit IgG antibody). Then, cells were washed with 1 × PBS three times. The fluorescence of the cells was acquired by microscopy (Nikon, Japan) and then analyzed.

### Western blotting assays

Transfected or infected GEFs were harvested and lysed in the cell lysis buffer. Equal amounts of the samples were then subjected to SDS-PAGE and analyzed the expression of the Rep1 protein using the rabbit anti-GPV Rep polyclonal antibody as the primary antibody. The expression of β-actin was detected using the mouse anti-β-actin monoclonal antibody as the primary antibody (mouse anti-β-actin monoclonal antibody) as loading control.

### Quantitative PCR (qPCR) analysis of viral genome

Virus samples were collected from a 12-well plate and freeze and thaw three times. Viral DNA was extracted using the TIANamp Virus DNA/RNA Kit (TIANGEN, Beijing, China) according to the protocol. The viral genome level was quantified by a qPCR method. Briefly, a pairs primers (forward primers, 5′-TGCCGATGGAGTGGGTAAT-3′; reverse primer, 5′-TGCCGATGGAGTGGGTAAT-3′) has been designed for qPCR to amplified the GPV genome from nt 4640 to 4760; the qPCR was conducted using the 2 × EvaGreen® qPCR pre-Mix (Innovagene, Hunan, China) by a real-time PCR System (Bio-Rad, USA). The PCR conditions were one cycle of 95 °C for 10 min, followed by 40 cycles of 95 °C 10 s, 59.4 °C for 30 s and 72 °C for 30 s.

### Phylogenetic analysis

Phylogenetic analysis was constructed using the MEGA 6.0 version. The phylogenetic tree based on Rep1 and VP1 genes was built by the neighbor-joining method with 1000 bootstrap replications as described previously.

## Results

### Phylogenetic analysis of RC16 isolate base on Rep1 and VP1

**Fig. 1:**
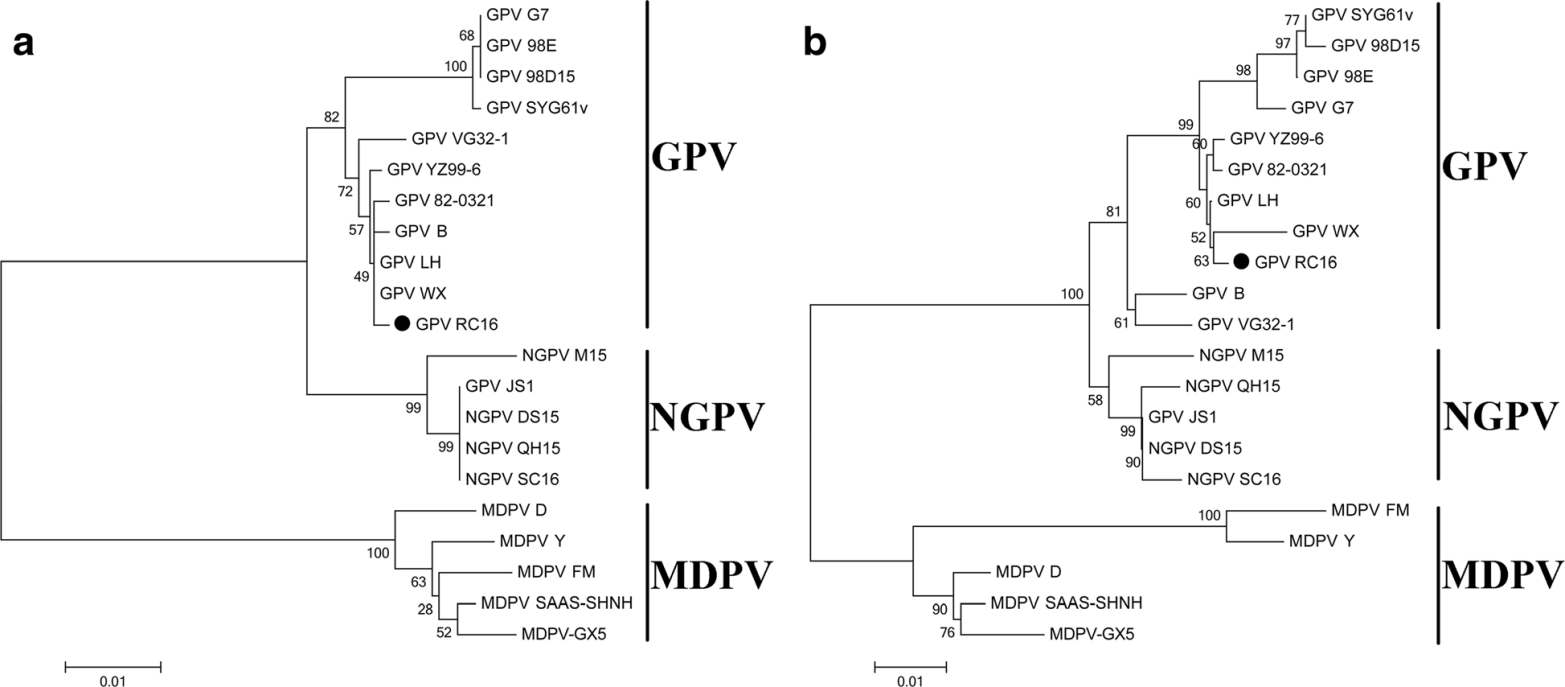
Phylogenetic analysis. The phylogenetic trees were constructed based on the amino acid sequence of Rep1 (**a**) and VP1 (**b**) by using the neighbor-joining method in MEGA 6.0

The full genome of GPV RC16 has been successfully cloned into the pMD19-T, and we have deposited the sequence of the full-length genome of the isolate in GenBank (Accession number: KY475562) [14]. To further determine the genetic diversity between RC16 and other GPV, MDPV, and NGPV isolates, phylogenetic trees of Rep1 and VP1 genes were constructed based on available sequences of waterfowl parvovirus from NCBI. The results of the phylogenetic analysis are shown in Fig. 1. The phylogenetic analysis based on the Rep1 and VP1 indicates that the RC16 isolate shows the closest genetic relationship with GPV strains (Fig. 1). Collectively, the RC16 isolate is proposed to be a member of the goose parvovirus.

### Construction and characterization of the full-length GPV RC16 clone

**Fig. 2:**
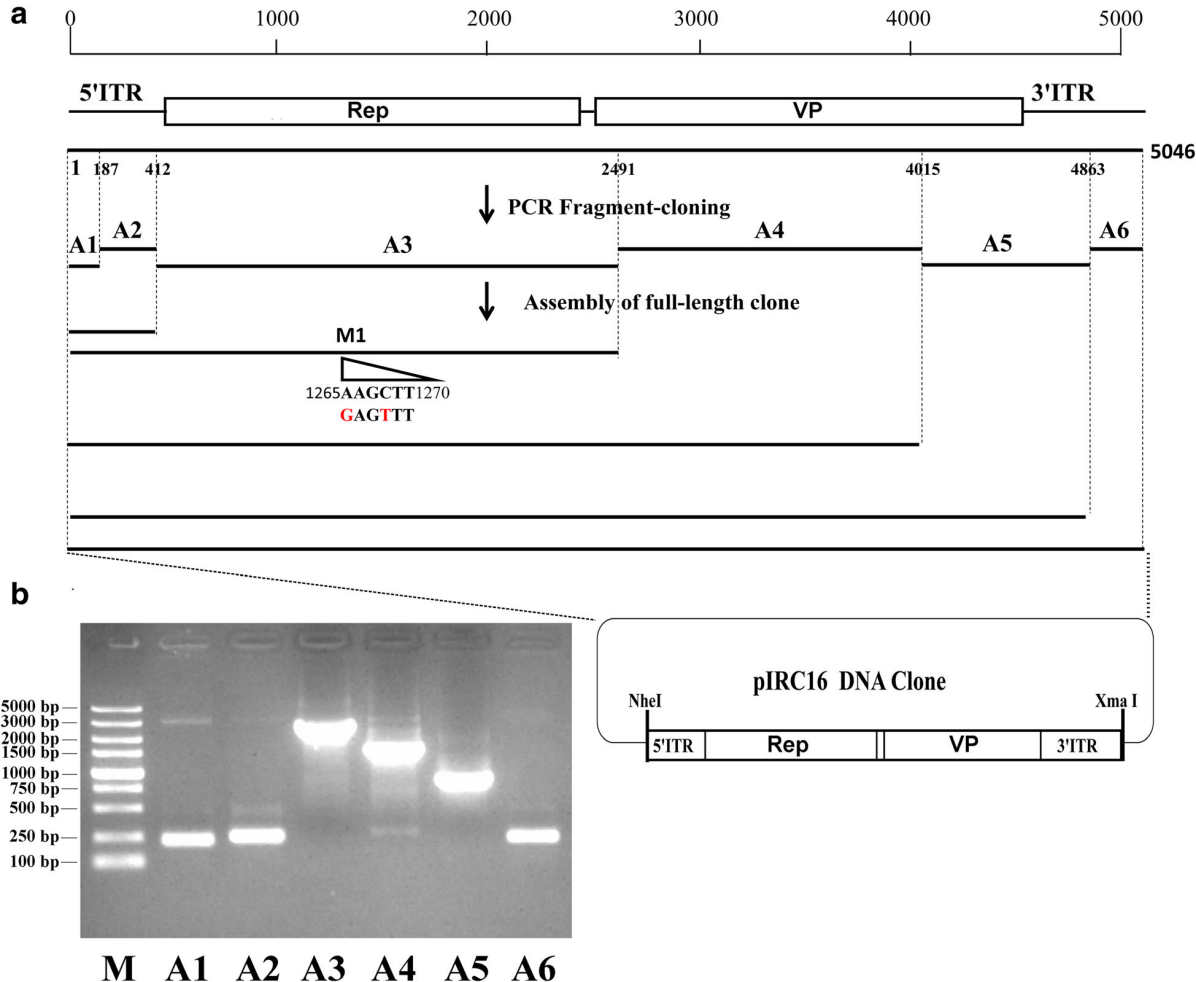
The construction of GPV RC16 reverse genetics strategy. **a** Six pair primers had been designed to amplify the complete genome based on the genome of the GPV YZ99–6 strain. The amplified fragments were A1, A2, A3, A4, A5, and A6, respectively. All fragments from A1 to A6 were orderly cloned into pACYC177. A genetic maker (M: AAGCTT→GAGTTT) was introduced into the site 1265 and 1268. **b** The amplified six fragments have been identified by Electrophoresis

As shown in Fig. 2a and described in Material and Method, every fragment was inserted into pMD19-T and sequenced before assembling, respectively. The full-length GPV RC16 clone was assembled by six fragments (Fig. 2b) and cloned into the low copy plasmid (pACYC177), and refer to this full-length clone as pIRC16 (Fig. 2a).

**Fig. 3:**
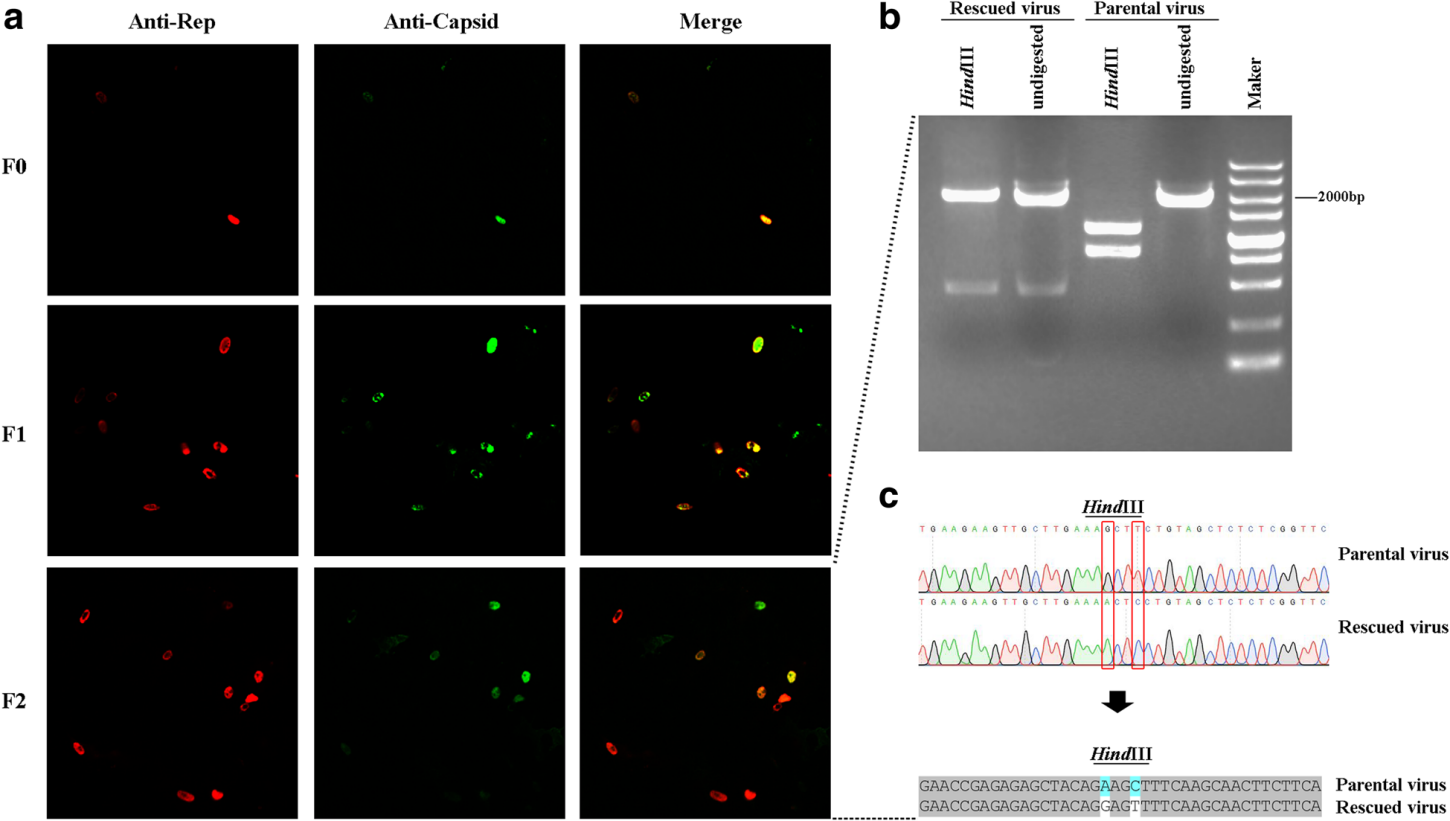
Identification of the recombinant GPV. **a** The expressions of Rep1 and capsid proteins had been identified at F0, F1, and F2 of pIRC16. **b** The amplified A3 from the rGPV RC16 F2 and the parental GPV RC16 had been digested by *Hind*III, while the A3 from GPV RC16 could be digested to two fragments of 1.2 kb and 0.8 kb by *Hind*III resulting in. **c** The amplified A3 had been sequenced and the genetic maker had been verified

The recombinant virus was rescued by transfection of pIRC16 into GEFs. Subsequently, the cells were harvested after 84 h (designated P0 viruses) and then blind passaged twice in GEFs. Meanwhile, the harvested cells were analyzed by WB using the anti-GPV Rep antibody, and the GPV Rep1 can be detected by WB (data not shown). Moreover, immunofluorescence analysis (IFA) was used to detect the expression of viral Rep and capsid proteins in F0, F1, and F2 in GEFs. As shown in Fig. 3a, the viral Rep and capsid proteins were detected at 120 h post-infection by IFA using rabbit anti-Rep polyclonal antibodies and mouse anti-GPV polyclonal antibodies. To discriminate the rescued virus from its parental strain, a genetic maker was introduced into the rescued virus, resulting in the deletion of *Hind*III within the region of Rep1 in rescued virus. As shown in Fig. 3b, the fragment amplified from the rescued virus and containing the region of Rep1 cannot be digested by *Hind*III, while the fragment amplified from the parental virus and containing the region of Rep1 can be digested by *Hind*III, resulting in a 1.2 kb and a 0.8 kb fragment (Fig. 3b). The results of sequencing covering the region of the genetic maker from the rescued virus shown the *Hind*III sites were deleted and the unimodal map was identified in mutant sites (1265A → G and 1268C → T) in the rescued virus (Fig. 3c). These results indicated that the rescued virus is from pIRC16.

Collectively, these results confirm that we have generated a full-length clone of GPV which can produce progeny virus in the transfected GEFs.

### GPV progeny virus produced by pIRC16 transfected cells is infectious

**Fig. 4:**
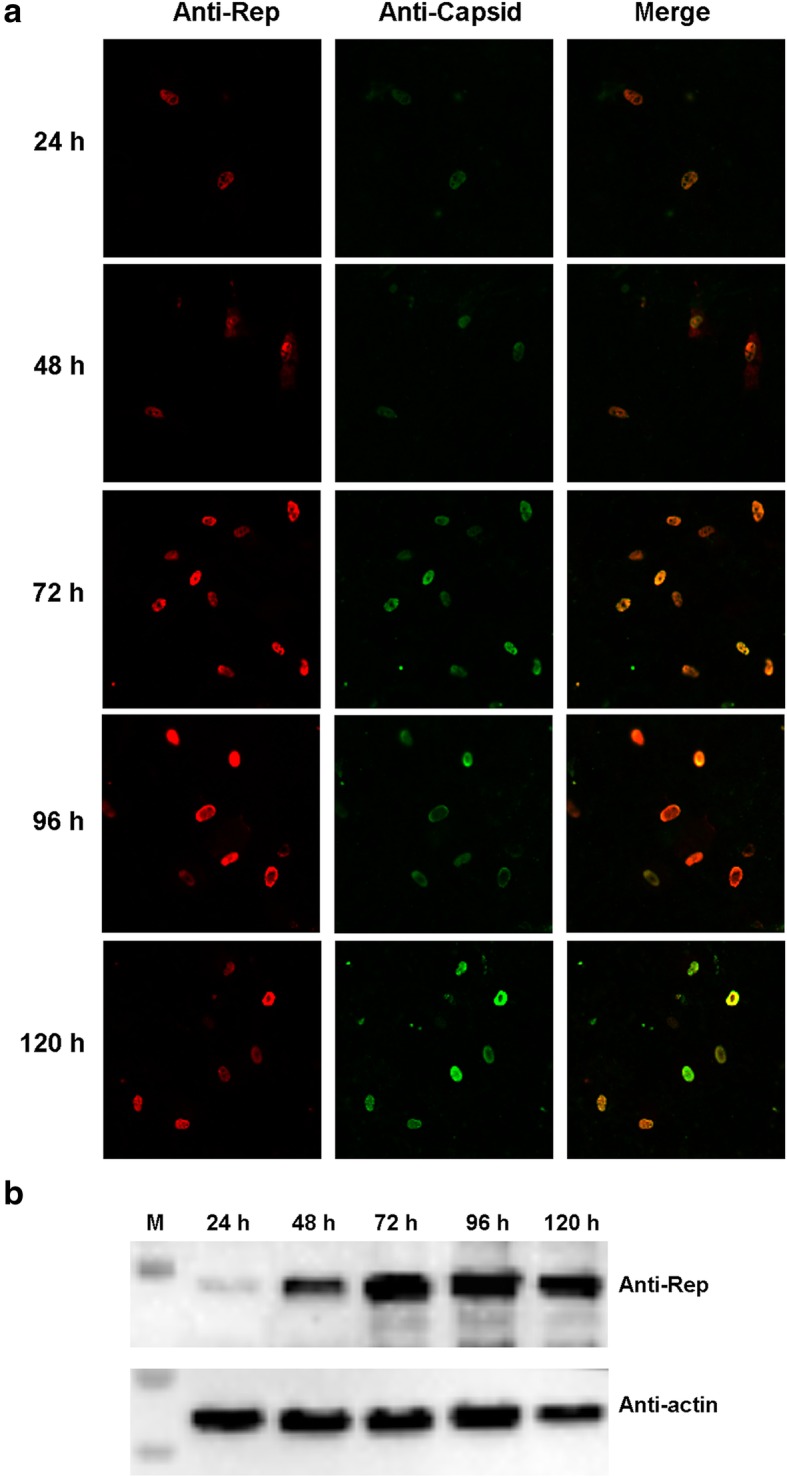
The recombinant GPV proliferated well in GEF. **a** IFA of GEF cells infected with the rGPV RC16 F2 as detected using the mouse anti-GPV polyclonal antibody and the rabbit anti-GPV Rep polyclonal antibody. **b** The expression of GPV Rep1 from the rGPV RC16 F2 was detected by WB using the rabbit anti-GPV Rep polyclonal antibody from 24 h to 120 h

No cytopathic effects were observed in rGPV RC16 F2 infected GEFs at 120 h PI (data not shown). However, positive signals were detected in rGPV RC16 F2 infected GEFs at 120 h PI by the rabbit anti-Rep polyclonal antibody (Fig. 3a). Subsequently, the F2 viruses from 24 h to 120 h were detected by IFA using the rabbit anti-Rep polyclonal antibody. As shown in Fig. 4a, the positive cells were detected by IFA using the anti-Rep antibody at 24 PI. The positive cells increased after 48 h PI. Furthermore, the expression of GPV Rep1 was also detected from 24 h to 120 h by WB using the anti-Rep antibody (Fig. 4b), suggesting that the rescued virus replicated well. Moreover, the genome numbers of F2 viruses from 24 h (10^7.27^copies/ml) to 120 h (10^8^ copies/ml) were detected by qPCR and the genome number of rescued virus continuously increased to a peak of 10^8^ copies/ml at 120 h.

Taken together, these results indicate that the GPV virions produced by pIRC16 transfection are capable of proliferation in GEFs.

### The 164 K, 165 K, and 167 K of VP1 are vital for proliferation of rGPV RC16 in vitro

**Fig. 5:**
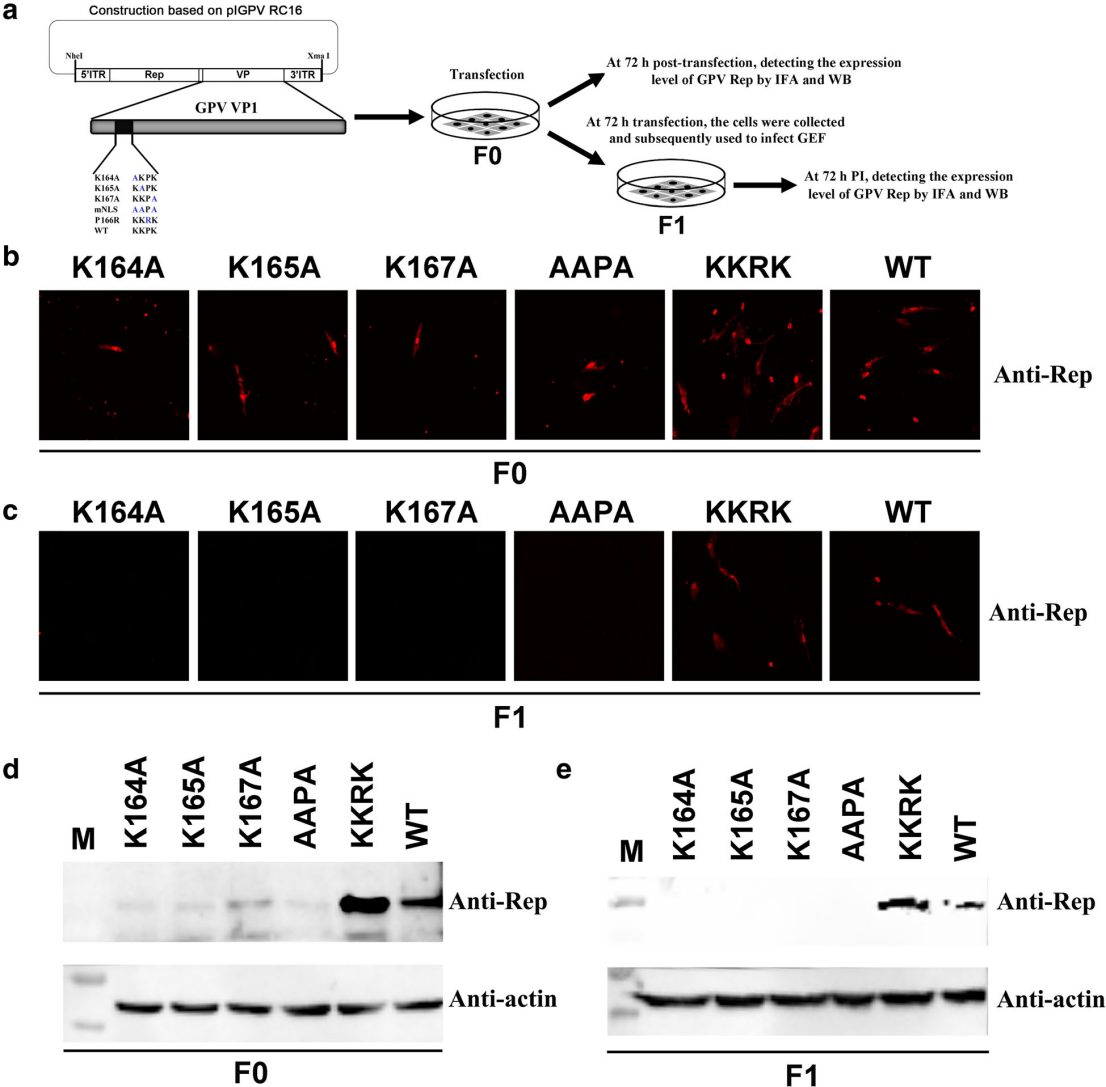
The 164 K, 165 K, and 167 K are vital for GPV proliferation. **a** Construction and transfection of pIRC16 K164A, pIRC16 K165A, pIRC16 K167A, pIRC16 AAPA and pIRC16 KKRK. On one hand, the cells were collected to detect the expression level of GPV Rep by IFA and WB at 72 h post-transfection. F0 represents the virus transfected pIRC16 K164A, pIRC16 K165A, pIRC16 K167A, pIRC16 AAPA, and pIRC16 KKRK respectively. On the other hand, the cells were collected to infect GEFs at 72 h post-transfection. F1 represents the virus from passaged F0. **b** and (**d**) At 72 h post-transfection, the GPV Rep of F0 was detected by IFA and WB using the rabbit anti-GPV Rep polyclonal antibody. **c** and (**e**) At 72 h PI, the GPV Rep of F1 was detected by IFA and WB using the rabbit anti-GPV Rep polyclonal antibody

**Fig. 6:**
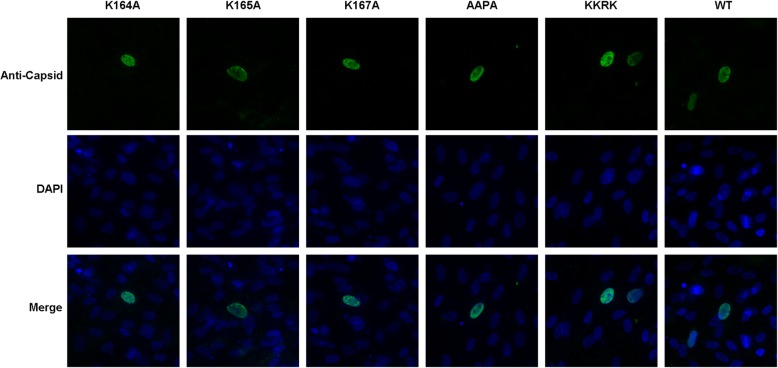
The GPV capsid with mutated 164 K, 165 K or 167 K of VP1 can enter into the nucleus. At 72 h post-transfection with pIRC16 K164A, pIRC16 K165A, pIRC16 K167A, pIRC16 AAPA, pIRC16 KKRK and pIRC16, the capsid protein could be detected in the nucleus by IFA using mouse anti-GPV capsid polyclonal antibody

In the previous study, the 164 K, 165 K, and 167 K were identified as the NLS key amino acids, which are vital for the transport of GPV VP1 into the nucleus [13]. Therefore, the NLS key amino acids were further studied based on pIRC16. And four NLS defect clones and one clone with 166 P to R within in GPV VP1 were constructed, the pIRC16 K164A, pIRC16 K165A, pIRC16 K167A, pIRC16 AAPA and pIRC16 KKRK respectively. All of them have been respectively transfected into GEFs to rescue virus. After 72 h PI, the GEFs were fixed and analyzed by IFA using anti-GPV Rep, and cells were harvested and analyzed by WB using the anti-GPV Rep, respectively. In line with the date of WB (Fig. 5d), the expression level of GPV Rep has been observed in rGPV RC16 K164A, rGPV RC16 K165A, rGPV RC16 K167A, rGPV RC16 AAPA by IFA, which is less than rGPV RC16 KKRK and rGPV-RC16 (Fig. 5b). Subsequently, the expression level of GPV Rep from its progeny virus has been observed by IFA and WB. However, the expression of GPV Rep hasn’t been observed in rGPV RC16 K164A, rGPV RC16 K165A, rGPV RC16 K167A, rGPV RC16 AAPA by IFA and WB (Fig. 5c and e). To exclude the mutant affects or inhibits the expression of GPV capsid, subsequently, the pIRC16 K164A, pIRC16 K165A, pIRC16 K167A and pIRC16 AAPA were respectively transfected into GEFs, and the expression of GPV capsid was identified by IFA. Importantly, expression and localization of GPV capsid into the nucleus have been observed after the transfection of pIRC16 K164A, pIRC16 K165A, pIRC16 K167A and pIRC16 AAPA (Fig. 6). Those results indicated that the 164 K, 165 K, and 167 K are fatal for the proliferation of GPV. However, all of them aren’t necessary for the accumulation of capsid into the nucleus during the viral life cycle, suggesting that the structural NLS may be required for transport of capsid into the nucleus.

## Discussion

In this study, we have identified the entire genome sequence of GPV RC16 and establish a PCR-based reverse genetics system with high fidelity for GPV RC16 strain. In the process of construction, we firstly used the high copy plasmid as the backbone. However, the plasmid containing intact ITR was unstable in *E. coli* SURE strain. The possible mechanism of instability of plasmids containing the intact ITR may be the palindromic sequence within ITR is difficult to amplify in the *E. coli* SURE strain. To avoid the deletion of palindromic sequence within ITR in the process of construction, the full-length genome was cloned into the low copy plasmid by ligation of all of the fragments from amplification of genome. Subsequently, the full-length plasmid was transformed into *E. coli* SURE strain. The SURE was cultured at 30 °C in 150 rpm. A reverse genetics system of human bocavirus was successfully established in this way [15]. Therefore, it is a novel method for the construction of infectious clones based on PCR in parvovirus.

Previously, an MDPV infectious clone was constructed by ligation of the MDPV double-strand DNA (dsDNA) into the plasmid by TA clone [16]. However, sequencing is difficult for the entire genome due to the palindromic sequence of ITR and whether obtaining an intact genome is unclear in the process of construction. A GPV infectious clone was constructed by restriction enzyme digestion and ligation of the GPV dsDNA [17, 18]. However, the viruses were rescued by goose embryo and the growth character of it is unclear in vitro [17, 18]. To further study the molecular mechanism of replication in vitro, the virus can be rescued in cells is important. In this study, the rescued virus is capable of proliferation in GEFs, suggesting that this method could be used in further study.

At present, although the live attenuated vaccines are widely used for controlling GPV, the goslings still highly suffered from GPV infection. More important, an outbreak of short beak and dwarfism syndrome disease in Cherry Valley duck in China is identified as an NGPV during 2015 [4, 5], which can cause low morbidity. Although the morbidity is low, the NGPV can lead to much economic loss in the duck industry due to the retarded growth of ducks infected. Genetic and antigenic analysis indicated that the NGPV is closely related to GPV [4, 19]. There is a high identity in the genome between GPV and NGPV, however, the host range and pathogenicity are different [20]. The construction of GPV infectious clone will provide a way to study the difference between GPV and NGPV.

In the previous study, the 164 K, 165 K and 167 K residues in GPV VP1 were identified as the key sites of GPV NLS, which were required for translocation of GPV VP1 into the nucleus of cells [13]. Moreover, a basic region (166PA**RKR**LN172) within AAV2 VP1, similar to GPV NLS sequence (160YPVV**KK**P**K**LTEE171), is required for early infection, especially delivery of its genome into the nucleus [21]. However, it is still unknown whether the key sites of GPV NLS affect the infectivity of GPV in the process of proliferation. The rGPV with mutated forms of each key site of NLS was assessed for its ability to proliferate in the lifecycle. Our data showed rGPV with mutation at 164 K, 165 K or 167 K of VP1 can’t proliferate in vitro. However, the transfection of the infectious clones containing the mutated 164 K, 165 K or 167 K resulted in the formation of the capsid in the nucleus. These data indicated that this NLS might be required for the delivery of viral genomes to the nucleus in initial infection but not essential for the transport of the assembled capsid into the nucleus in the late steps of infection. Therefore, the rGPV with NLS defect is unable to complete its infectious cycle, strongly suggesting that the 164 K, 165 K, and 167 K are essential for infectivity in the early infection phase. The structural NLS has been identified in porcine parvovirus and minute virus of mice, which is a member of the *Protoparvovirus* genus of the *Parvovirnae* subfamily of the *Parvoviridae* family, and depends on the correct folding of capsid protein [22, 23]. Importantly, our study shows that the capsid with mutated NLS can target into nucleus, suggesting the GPV capsid may depend on a structural NLS to target into the nucleus.

## Conclusions

In conclusion, we establish a reverse genetics system for GPV RC16 based on PCR, which can be applied to other parvovirus. The rescued viruses are capable of proliferation in vitro. The 164 K, 165 K and 167 K of VP1 are vital for the proliferation of GPV in vitro. This system will enable us to gain a better understanding of the mechanism of GPV replication and pathogenesis.

## Data Availability

All data generated or analyzed during this study are included in the published article.

## Abbreviations

BR: Basic region
GEFs: Goose embryo fibroblasts
GPV: Goose parvovirus
IFA: Immunofluorescence analysis
ITR: Inverted terminal repeats
MDPV: Muscovy duck parvovirus
NGPV: Novel goose parvovirus
NLS: Nuclear localization signal
ORF: Open reading frames
qPCR: Quantitative PCR
Rep1: Replication protein 1
